# Miniscalpel-Needle Treatment Is Effective for Work-Related Neck and Shoulder Musculoskeletal Disorders

**DOI:** 10.1155/2016/5760240

**Published:** 2016-06-13

**Authors:** Shuming Li, Tong Shen, Yongshan Liang, Bo Bai, Ying Zhang

**Affiliations:** ^1^Department of Rehabilitation Medicine, First Affiliated Hospital of Guangzhou Medical University, No. 151, Yanjiang West Road, Guangzhou, Guangdong 510120, China; ^2^Department of Orthopedics Medicine, First Affiliated Hospital of Guangzhou Medical University, No. 151, Yanjiang West Road, Guangzhou, Guangdong 510120, China; ^3^Department of Orthopedics Medicine, Beijing Fengsheng Special Hospital of Traditional Medical Traumatology and Orthopaedics, No. 306, Fuchengmen Street, Beijing 100034, China; ^4^Guangdong Key Laboratory of Orthopaedic Technology and Implant Materials, No. 151, Yanjiang West Road, Guangzhou, Guangdong 510120, China

## Abstract

*Background.* Work-related musculoskeletal disorders (MSDs) are a group of painful disorders of muscles, tendons, and nerves, such as neck and shoulder MSD. This study was designed to use miniscalpel-needle (MSN) technique as an intervention for work-related MSDs.* Methods.* Thirty-one patients with work-related MSDs and 28 healthy subjects were enrolled as controls in this study. The MSD symptoms of each patient were assessed by visual analog scale (VAS) and neck disability index (NDI). Blood samples were collected from control subjects and MSD patients before and after treatment. Serum levels of C-reactive protein (CRP) and tumor necrosis factor (TNF) were measured using ELISA.* Results.* Prior to MSN treatment, serum levels of CRP and TNF were significantly higher in the MSD patients than the healthy controls. Serum CRP levels correlated with VAS and NDI scores, and serum TNF levels correlated with NDI scores. Compared to pretreatment, VAS and NDI scores were significantly lower in MSD patients after MSN treatment, while serum CRP and TNF levels were significantly lower compared with the healthy control levels.* Conclusions.* Our results indicate that MSN may be an effective intervention for work-related MSDs and be associated with lower serum levels of inflammatory biomarkers.

## 1. Background

The US Department of Labor defines work-related musculoskeletal disorders (MSDs) as injuries or disorders of the muscles, nerves, tendons, joints, cartilage, and spinal discs associated with exposure to risk factors in the workplace [1]. MSDs have become common and physically disabling occupational health problems in modern society and shown an increasing trend [2, 3]. Aggravated MSDs have been associated with high levels of inflammatory mediators. For example, a study of rats subjected to long-term performance of a highly repetitive, low-force task to determine an association between grip strength and muscle-tendon responses found that C-reactive protein (CRP) and tumor necrosis factor (TNF) could serve as serum biomarkers of work-related MSDs [4].

Force-repetition has consistently been shown to increase the risk of MSDs; low- and high-force repetitive tasks, respectively, induce a modest or rapid increase in risk [5]. In addition, a significant increase in serum concentration of TNF was observed in rats performing highly repetitive, high-force tasks [6–10], and serum concentrations of several inflammatory biomarkers were related to signs and symptoms in patients with newly developed upper extremity MSDs [11–13]. Furthermore, serum concentrations of inflammatory biomarkers were higher in MSD patients than in healthy controls and correlated with pain intensity [14, 15]. Overall, these findings suggest that inflammatory responses are associated with MSD development, and inflammatory mediators may serve as biomarkers of the effectiveness of therapeutic interventions for MSD [11, 13, 14].

The miniscalpel-needle (MSN) is a medical instrument similar to the acupuncture needle [16–20]. Treatment with MSN has been reported to relieve the symptoms of cervical myofascial pain syndrome without severe side effects [16, 18, 19]. These results suggest that MSN may be a promising treatment for work-related neck and shoulder MSDs.

In this study, a population of nurses and computer engineers with worked-related MSDs and healthy controls were recruited. MSD patients received MSN treatment, while healthy subjects without any treatment served as control group. The effectiveness of MSN was measured by visual analog scale (VAS) and neck disability index (NDI), which were suggested by previous study [19]. We hypothesized that (1) MSN treatment would effectively relieve the severity of MSD symptoms in patients and (2) serum levels of CRP and TNF might be correlated with work-related MSD symptoms.

## 2. Methods

### 2.1. Patients

This prospective cohort study was conducted from December 2012 to July 2013 at the Department of Rehabilitation Medicine, First Affiliated Hospital of Guangzhou Medical University, after the approval by the hospital's Ethics Committee (Trial Registration: 2012-47).

Nurses and nursing aides suffering from work-related MSDs have been widely investigated [21, 22]. Information technology has revolutionized economies throughout the world, even more dramatically in China. Computer work is widely perceived as a new risk factor for MSDs, which have been frequently diagnosed as occupational problems in China and other countries [23–26]. In current study, we focused on the nurses and computer engineers who are at high risk for work-related MSDs. Nurses and computer engineers with work-related neck and shoulder MSDs were recruited from the outpatient clinic. X-ray was performed to exclude spinal disorders. Among 43 patients screened, 12 were excluded and the remaining 31 patients were assigned to the MSD group of the study. The healthy control group consisted of 28 nurses and computer engineers with no MSD-related symptoms, recruited through advertisements in the hospital.

All participants provided informed consent and they were advised to avoid any additional treatments during the study, including western medicine, physical therapy, and acupuncture. Every participant was allowed to withdraw from the study at any time, to receive other treatment or for any other reason.

### 2.2. Inclusion Criteria

Patients were included if they had neck or shoulder pain, or both, that was due to work-related MSD; were 18 to 50 years old; had symptoms lasting less than 3 months; had pain of the neck or shoulder > 4 on a visual analog scale (VAS; 0 corresponding to no pain, 10 to the worst possible pain experienced by that individual) [27, 28]; and the intensity of pain of other body parts was VAS < 3.

### 2.3. Exclusion Criteria

Patients were excluded if they had neck pain caused by a neurological or rheumatic disease; had contraindications to MSN treatment such as infection, spinal fracture, osteoporosis, pregnancy, or a bleeding disorder; had psychiatric disorders or other health problems requiring medication; had smoked or had cold or other infections during the previous month; or had previous MSN treatment.

Elevated CRP levels have been associated with age, the use of certain drugs, obesity, smoking, systemic diseases (e.g., arthritis, diabetes, and inflammatory, cardiovascular, and metabolic diseases), and certain lifestyles [29–36]. Therefore, we excluded subjects older than 50 years, to avoid potential masking of the effects of MSN.

### 2.4. Outcome Measures

Pain intensity was measured by VAS [27, 28]. Neck disability was measured using the Chinese version of the neck disability index (NDI), which is a 10-item, 50-point index assessing the aspects of daily function in patients with neck pain [37, 38].

Serum levels of CRP and TNF were measured by enzyme-linked immunosorbent assay (ELISA). The blood samples were allowed to stand for 15 min and then centrifuged for 15 min at 2000 ×g. Serum samples were collected and stored at −80°C. Serum concentrations of CRP and TNF were measured by commercial kits (Human CRP Quantikine ELISA, DCRP00, and Human TNF Quantikine HS ELISA, HSTA00D; R&D Systems, Minneapolis, MN, USA) in accordance with the manufacturer's protocol.

### 2.5. Procedures

Prior to MSN treatment, VAS and NDI were measured by a qualified physical therapist, and blood was collected from patients in the MSD group and the healthy controls.

MSN treatment was performed as previously described [16, 17]. Briefly, patients sat in a chair with arms at their sides. One or two tender points on the neck or shoulder were located by palpating with the tip of the thumb and marked. At each mark, a sterilized MSN (0.80 mm in diameter and 60 mm long, Huaxia Acupotomology Medical Equipment Factory, Beijing, China) was inserted into the tender point vertically to a depth of 20–30 mm, parallel to the spine. When the tip of the MSN reached the area of injury, the doctor likely felt heaviness or resistance, and the patient often had a strong needling sensation consisting of distention, soreness, or heaviness. A strong needling sensation at the tender point indicated correct placement of the needle. The MSN was moved up and down without rotation 3 to 5 times to release the pain and withdrawn when the strong needling sensation decreased. The hole made by the needle was covered by a simple adhesive bandage for one day. After treatment, patients were observed for 30 min for possible adverse reactions.

After MSN treatment, patients returned to their previous workplaces and exposure to the same risk factors. Two weeks after MSN treatment, VAS and NDI were measured and blood was collected again in MSD patients at the hospital outpatient clinic. All patients returned to the clinic and completed the follow-up. The same physical therapist evaluated VAS and NDI in the first and second visit for each patient and was blind to the MSN treatment.

### 2.6. Sample Size

The statistical power (1-*β*) was defined as 0.9 to reject a false null hypothesis in this study. When estimating the sample size for analyzing the VAS difference (primary aim of current study) in MSD patients between pre- and posttreatment, we assumed that the standard deviation equals 3 according to the literature [39]. A decrease of 2 points on VAS represents a minimum clinically relevant improvement [40, 41]. In order to detect a significant difference with power of 0.90 and probability of <0.05 (two-tailed), a sample size of 26 patients was required. Giving a loss to follow-up of 20%, we aimed to include 31 subjects in this study.

### 2.7. Statistical Analysis

Normally distributed variables are reported as the mean ± standard deviation and nonnormally distributed variables as the median (range). Categorical variable (gender and occupation) was compared using chi-squared test. Age and body mass index (BMI) were assessed using independent-sample *t*-test. Serum CRP and TNF concentrations in the control group and pre-/posttreatment patients were compared using independent-sample *t*-tests. Serum CRP and TNF levels of the MSD group before and after MSN treatment were compared using paired Student's* t*-tests. The VAS and NDI scores of the MSD group before and after MSN treatment were compared using the Wilcoxon matched-pairs signed rank test. Correlations of serum CRP and TNF levels with VAS and NDI scores were assessed by Spearman's rank analysis. All statistical analyses were performed using SPSS 17.0 (SPSS, Chicago, IL, USA). A two-tailed *P* < 0.05 was considered statistically significant.

## 3. Results

The subjects in control groups and MSD patients were not significantly different in the characteristics, in terms of age, gender, BMI, and occupation (Table 1). The duration of neck and shoulder symptoms in the MSD group ranged from 1 to 3 months. No MSD-related symptoms were found in the healthy controls.

**Figure 1:**
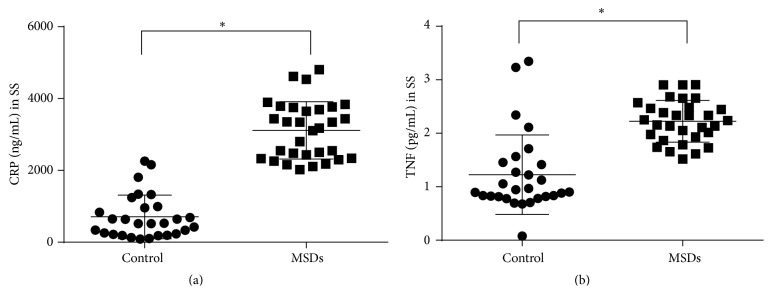
Serum levels of CRP and TNF in the subjects of healthy control and MSD before treatment group. SS: systemic serum. ^*∗*^ *P* < 0.05 examined by independent-sample *t*-tests.

**Figure 2:**
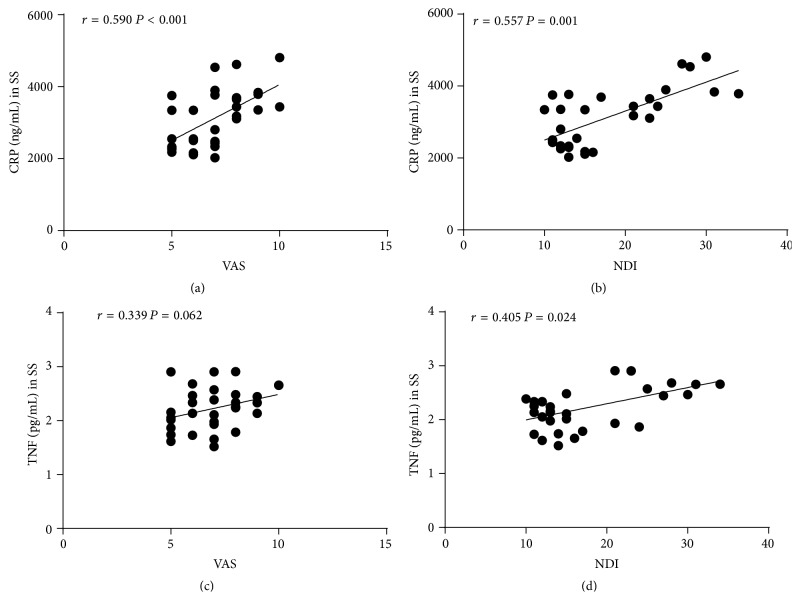
Correlations of serum CRP and TNF levels with VAS and NDI before treatment in the MSD group. Correlations between (a) serum CRP level and VAS; (b) serum CRP level and NDI; (c) serum TNF level and VAS; and (d) serum TNF level and NDI. Results are shown as Spearman's rank correlation coefficients. SS: systemic serum.

Before MSN treatment, serum CRP and TNF levels of the MSD patients were significantly higher than those of the control group (3115.0 ± 799.6 ng/mL versus 705.8 ± 608.8 ng/mL for CRP, 2.225 ± 0.391 pg/mL versus 1.225 ± 0.742 pg/mL for TNF; *P* < 0.05 examined by independent-sample *t*-tests) (Figure 1). Moreover, Spearman's rank correlation coefficients showed that serum CRP levels significantly correlated with scores of VAS (*r* = 0.590, *P* < 0.001) and NDI (*r* = 0.577, *P* = 0.001) (Figure 2) in MSN patients before treatment. Serum TNF levels positively correlated with NDI score (*r* = 0.405, *P* = 0.024), but not VAS score (*r* = 0.339, *P* = 0.062). These results indicated that the serum CRP and TNF level might reflect the severity of MSD symptoms.

**Figure 3:**
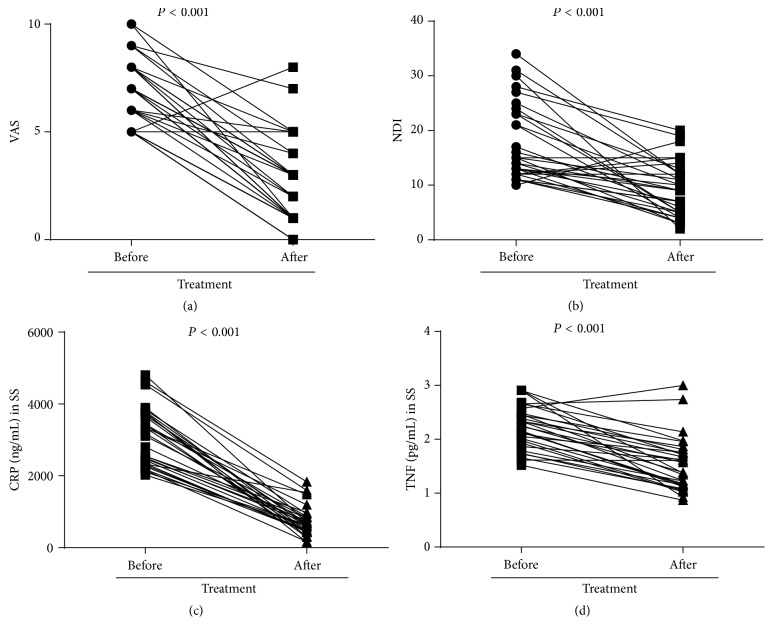
VAS, NDI, and serum CRP and TNF levels before and two weeks after MSN treatment in MSD patients. (a) VAS and (b) NDI scores of the MSD group before and two weeks after MSN treatment were compared using the Wilcoxon matched-pairs signed rank test. Serum (c) CRP and (d) TNF levels before and two weeks after MSN treatment were compared using paired Student's *t*-tests. SS: systemic serum.

**Figure 4:**
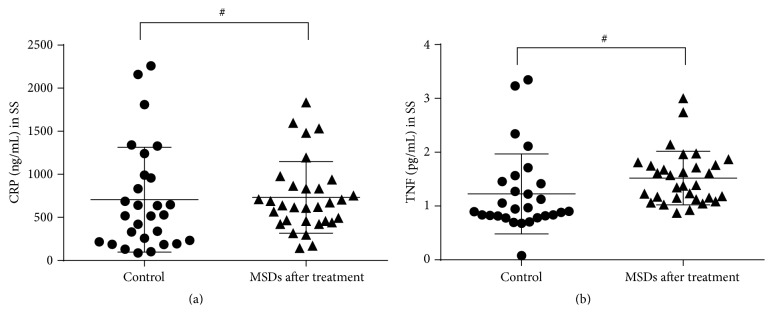
Serum levels of CRP and TNF in the subjects of healthy control and MSD after treatment group. SS: systemic serum. ^#^ *P* > 0.05 examined by independent-sample *t*-tests.

All 31 patients with MSDs completed the MSN treatment and the follow-up. When compared to pretreatment, MSD patients after treatment showed significant improvement in their MSD symptoms, indicated by the decrease in VAS (Figure 3(a)) and NDI (Figure 3(b)) scores (*P* < 0.001 tested by the Wilcoxon matched-pairs signed rank test). Moreover, serum CRP (Figure 3(c)) and TNF (Figure 3(d)) levels in MSD patients after MSN treatment also decreased significantly in comparison with pretreatment (*P* < 0.001 tested by paired Student's *t*-tests). We further compared the serum levels of CRP and TNF in MSD patients after MSN treatment with those of healthy control subjects and found no differences (731.5 ± 415.9 ng/mL versus 705.8 ± 608.8 ng/mL for CRP, 1.519 ± 0.497 pg/mL versus 1.225 ± 0.742 pg/mL for TNF; *P* > 0.05 by independent-sample *t*-tests) (Figure 4). This indicated that MSN treatment not only relieved the severity of symptoms in MSD patients but also decreased the serum CRP and TNF levels to the control subject levels.

**Figure 5:**
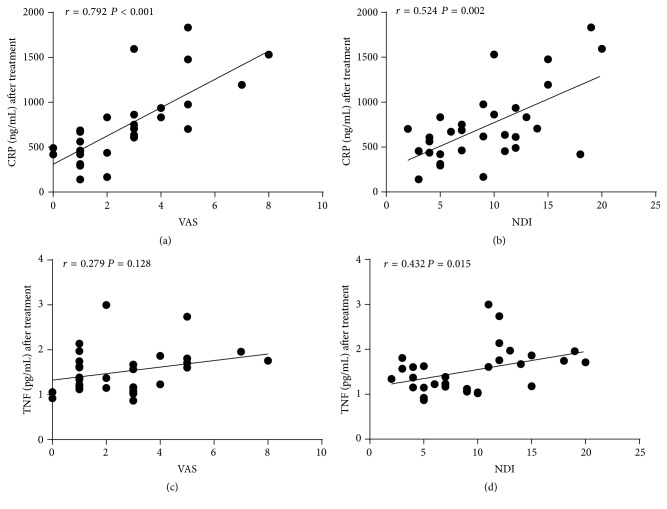
Correlations of serum CRP and TNF levels with VAS and NDI after treatment in the MSD group. Correlations between (a) serum CRP level and VAS; (b) serum CRP level and NDI; (c) serum TNF level and VAS; and (d) serum TNF level and NDI. Results are shown as Spearman's rank correlation coefficients. ASS: systemic serum after treatment.

In order to further examine whether serum CRP or TNF levels were associated with the severity of MSD symptoms, we tested the correlations between serum CRP or TNF levels with VAS or NDI scores in MSD patients after MSN treatment by Spearman's rank analysis (Figure 5). It was found that CRP level significantly correlated with VAS (*r* = 0.792, *P* < 0.001) and NDI (*r* = 0.524, *P* = 0.002) score. Serum TNF levels positively correlated with NDI score (*r* = 0.432, *P* = 0.015), but not VAS score (*r* = 0.279, *P* = 0.128). The result is in agreement with the correlation test in MSD patients before MSN treatment, which further supports our hypothesis that serum CRP and TNF levels are associated with VAS and NDI scores.

## 4. Discussion

Work-related MSDs are a serious health problem in modern society and show an increasing trend. To the best of our knowledge, this is the first study to investigate the effectiveness of MSN treatment for patients with work-related MSDs. Our results showed that MSN treatment appeared to lessen the symptoms of MSD in our patients and was associated with decreased serum levels of inflammatory biomarkers.

MSN treatment essentially combines microinvasive surgery with acupuncture. Similar to microinvasive surgery, MSN may detach taut bands in patients with myofascial pain syndrome, relax compressed nerves and vessels, and improve local microcirculation [42, 43]. Similar to acupuncture, MSN may have analgesic effects on the spine and modulate the expression of transmitters and cytokines related to hyperalgesia [44, 45].

There was a significant improvement in both the subjective and objective measures of the VAS and NDI after the MSN treatment was administered to patients in the MSD group, and these correlated with reduced serum CRP and TNF levels. MSN treatment has been reported to provide greater pain relief in patients with cervical myofascial pain syndrome than either trigger point injection or acupuncture, with no severe adverse side effects [16, 18, 19]. However, these outcomes could not be assessed objectively, which limits the application of MSN.

CRP is an acute-phase marker of low-grade inflammation [14]. Consistent with previous studies [11, 14, 15], we observed that baseline serum CRP levels in MSD patients were significantly higher than those of the healthy control group, and serum CRP levels correlated with both VAS and NDI. The higher serum CRP levels of the MSD patients might have been due to local inflammation of injured tissue. After the MSN treatment, serum CRP levels significantly decreased and still showed strong correlations with VAS and NDI. In addition, posttreatment serum CRP levels were comparable to those of the control group, suggesting that the inflammation in the injured tissue was alleviated two weeks after MSN treatment. Thus, serum CRP levels may be a biomarker to evaluate the effects of MSN on MSD patients.

TNF is produced by injured cells and immune cells and regarded as a proinflammatory cytokine [46]. In this study, serum TNF levels were higher in MSD patients than in the healthy controls and correlated with NDI scores. This suggests an association between work-related MSDs and low-grade inflammation. Furthermore, in other studies, serum TNF levels were higher in animals and patients with MSDs induced by repetitive stress [11, 12, 47] and moderately correlated with MSD severity [11]. However, another study reported no correlation between serum TNF levels and pain intensity [14]. In the current study, we also found that serum TNF levels did not correlate significantly with VAS score. Nevertheless, two weeks after MSN treatment the serum TNF levels of MSD patients significantly decreased to levels comparable to those of the control group. Serum TNF levels significantly and positively correlated with NDI scores.

Interestingly, we found that serum CRP levels correlated more strongly with VAS and NDI scores than serum TNF levels. This is in agreement with other studies showing that CRP is more strongly related to functional impairments and sensory measures [11, 48]. Nevertheless, further studies are needed to determine whether serum CRP levels could be used to evaluate the effectiveness of MSN treatment for MSD.

There are several limitations in this study. First, the lack of a placebo MSD control group may be a source of bias. A control group of healthy subjects only helps describe the baseline characteristics of the population enrolled. However, a placebo control group may be unethical under these conditions. During this study, the MSD patients continued to be exposed to repetitive task at their workplaces, leading to a chronic inflammatory response due to continued demands on injured tissue. This could exclude the possibility of spontaneous recovery without any treatment [5, 10, 12]. We also ruled out a control MSD group receiving conservative treatments. MSN treatment is minimally invasive surgery. Most of the work-related MSD patients in the study had received certain conservative treatments, such as physical therapy, postural intervention, medicine, hot pack, massage, or stretch exercise before MSN treatment [16, 18–20]. The patients who failed in conservative treatments chose the MSN treatment in the outpatient clinic. The Wilcoxon matched-pairs signed rank test in MSD patients before and two weeks after MSN treatment did show the significant decrease in VAS and NDI scores.

Second, the subjects in MSD group were recruited from the outpatient clinic, while the subjects in the healthy control group were recruited via the hospital advertisement. As they were obtained from different populations, they may not be representative. This is our sampling concern.

Third, several studies reported that MSN treatment was effective in relieving chronic neck pain, but the proper tender points were crucial to the success of the treatment [16, 18, 19]. In MSN treatment, the accuracy of the needle placement highly relied on patient feedback of a strong needling sensation and the technical skill of the physician. Although the physician has been well trained and the strong needling sensation at the tender point could indicate correct placement of the needle, the position to perform the MSN treatment is still variable. Recently, ultrasound guidance has been shown to enhance the accuracy of the needle placement and improve the performance of MSN effectively [19]. Further study with an advanced method to guide the placement of MSN in an accurate position, for example, ultrasound, might be highly desirable. Another limitation is that only serum CRP and TNF levels were measured, and further investigations are needed to examine other proinflammatory cytokines such as IL-1 and IL-6.

## 5. Conclusions

We found that serum levels of the inflammatory biomarkers CRP and TNF correlated with the severity of MSD symptoms. MSN appears to be effective for treating work-related neck and shoulder MSDs and was associated with decreased serum levels of inflammatory biomarkers.

## Figures and Tables

**Table 1:** The characteristics of the subjects in the MSD and control groups.

	MSD			Control			*P* value
Occupation	Computer engineer *n* = 23	Nurse *n* = 8	Total *n* = 31	Computer engineer *n* = 16	Nurse *n* = 12	Total *n* = 28	0.213
Age, y (mean ± SD)	40.2 ± 12.7	39.1 ± 9.9	39.9 ± 11.9	36.8 ± 10.9	36.5 ± 10.0	36.7 ± 10.3	0.270
Gender (F/M)	14/9	8/0	22/9	9/7	12/0	21/7	0.728
BMI, kg/m^2^ (mean ± SD)	25.0 ± 5.0	21.5 ± 2.2	24.1 ± 4.6	23.1 ± 3.6	23.0 ± 3.5	32.1 ± 3.5	0.368
Duration, m [median (range)]	3 (1, 3)	1.5 (1, 3)	3 (1, 3)				
VAS [median (range)]	7 (5, 10)	6.5 (5, 10)	7 (5, 10)				
NDI [median (range)]	15 (11, 31)	13.5 (10, 34)	15 (10, 34)				

Normally distributed variables are reported as the mean ± standard deviation and nonnormally distributed variables as the median range. Categorical variable (gender and occupation) was compared using chi-squared test. Age and BMI were assessed using independent-sample *t*-test.

## Abbreviations

MSDs: Musculoskeletal disorders
MSN: Miniscalpel-needle
CRP: C-react protein
TNF: Tumor necrosis factor
VAS: Visual analog scale
NDI: Neck disability index
ELISA: Enzyme-linked immunosorbent assay
BMI: Body mass index
SS: Systemic serum.
